# Biological and Clinical Aspects on the Treatment of Schizophrenia and Related Disorders: New Challenges

**DOI:** 10.3390/healthcare10091806

**Published:** 2022-09-19

**Authors:** José Antonio Monreal, Alexandre González-Rodríguez

**Affiliations:** 1 Department of Mental Health, Mutua Terrassa University Hospital, Fundació Docència I Recerca Mutua Terrassa, University of Barcelona (UB), CIBERSAM, 08221 Terrassa, Spain; 2 Institut de Neurociències, Universitat Autònoma de Barcelona (UAB), 08221 Terrassa, Spain

For several decades, it has been postulated that dopaminergic pathways explain the neurobiology of schizophrenia, the biological underpinnings of treatment responses and the main mechanisms of action of antipsychotics [1]. The dopamine hypothesis explains how the vast majority of antipsychotics exert their positive effects in schizophrenia; however, other neurotransmitter pathways seem to explain how these drugs work on the brain [2]. Glutamatergic and serotonergic neurotransmitter systems have also been implicated, as have other biological compounds (e.g., hormones, inflammatory markers, etc.).

Patients with schizophrenia exhibit marked positive and negative symptoms, cognitive and affective symptoms and suicidal ideation and behavior [3]. In many cases, social and occupational deficits are present, and they remain therapeutic challenges. The associations between neurotransmitter systems and other biological pathways with symptom domains have been investigated in recent decades. Although antipsychotics are considered the gold standard in the treatment of schizophrenia and related disorders [4], preclinical and clinical studies report that almost one-third of patients with schizophrenia fail to respond to standard antipsychotic treatment. Patients are thus classified as responders, non-responders, or refractory. In treatment-resistant cases, clozapine can be indicated, as well as its combination with other therapies, for instance, psychological interventions [5]. Recent studies have indicated that adding cognitive remediation, especially coping and compensatory skills, to employment support services may improve global functioning in schizophrenia [5].

The present Special Issue aims to summarize new evidence on the roles of biological systems interacting with dopamine and other neurotransmitters implicated in the occurrence of psychotic symptoms and treatment responses in schizophrenia and related disorders. The roles of stress hormones and other hormonal compounds in clinical presentation (phenotype), physical health and responses to treatment will be explored. Evidence for the effectiveness of pharmacological and non-pharmacological interventions to treat schizophrenia and related disorders is also welcome, with a special emphasis on treatments targeting emotions, cognitive symptoms and suicidal behavior. The impact of physical health on psychotic symptoms and vice versa, and the risk of suicide, are also topics of interest.
